# Pulmonary Hypertension: A Personal Journey

**DOI:** 10.1002/pul2.70047

**Published:** 2025-02-03

**Authors:** Lewis J. Rubin

**Affiliations:** ^1^ Department of Medicine University of California San Diego School of Medicine La Jolla California USA; ^2^ Columbia University Vagelos College of Physicians and Surgeons New York New York USA

My first clinical experience with Pulmonary Hypertension occurred when, as a resident at Duke University Hospital in the late 1970's, a patient with “primary pulmonary hypertension (PPH)” was admitted with right heart failure. The patient, Mr. C., was a tobacco farmer from rural North Carolina. He was a pleasant man with a grade school education whose family had been farmers for generations, and likely slaves before then. He had no children, and his wife was deeply concerned about his health. Remembering nothing about pulmonary hypertension from medical school, I reached for the standard Textbook of Medicine: The section on *Primary Pulmonary Hypertension* was quite brief, consisting of two sentences stating that the cause was unknown, there was no treatment, and the afflicted patients died shortly after diagnosis from right heart failure.

I was intrigued by this rare and mysterious disease, so I sought the guidance of Robert H. (Jess) Peter, MD, a cardiologist at Duke who was regarded by the house staff as an outstanding clinician and educator. Jess gave me some published papers to read and introduced me to the Duke cardiology database—a remarkable resource about all patients with cardiovascular disease seen at this medical center.

I discharged Mr. C after aggressive diuresis and planned to see him in the clinic for follow‐up. I had read a great deal about the state of knowledge regarding the pulmonary circulation and contemporary treatment of heart failure, including the then “novel” concept of afterload reduction for left heart failure [1]. Somewhat naively, I postulated that this approach may be useful to treat pulmonary hypertension and right heart failure. When I approached Jess Peter to discuss this idea, he was enthusiastic and suggested that I write a protocol for submission to the Institutional Review Board for a study with hydralazine, a systemic vasodilator being used to treat systemic hypertension and left heart failure, in patients with PPH.

I identified three other PPH patients in the database, and both Mr. C and the others were eager to participate. Soon thereafter, I was in the cardiac catheterization laboratory and, under the guidance of Jess Peter and the other cardiologists, learning the science and methodology of invasive hemodynamics, particularly in the setting of pulmonary vascular disease. We evaluated the four PPH patients at rest and during upright bicycle exercise before and 4–6 months after treatment with oral hydralazine. The patients seemed to improve clinically, and the hemodynamic effects of therapy appeared beneficial. We published the results in a lead article in the New England Journal of Medicine [2] and the focus of my career became sharply defined.

A year later, I was called to the Emergency Department: Mr. C had been brought in with severe dyspnea and confusion and had died after experiencing a cardiopulmonary arrest. While I was fully aware that my new “treatment” for PPH was far from a cure, I was saddened and disappointed by Mr. C's death, even though I had observed him deteriorate over the last few clinic visits. Mr. C's wife was deeply affected by his death, and I tried to console her. I completed the death certificate, listing Primary Pulmonary Hypertension as the cause of death, and returned to my residency work and interest in the pulmonary circulation.

In the interim, experimental studies had shown that hydralazine might exert its vasodilatory effects by increasing the production of prostaglandins [3]. Fortuitously, I met with Dr. Michael Frosolono from Burroughs Welcome Pharmaceuticals in 1981 to discuss the potential of prostaglandin I_2_ (Prostacyclin), the most potent vasodilator prostaglandin, as a possible treatment for PPH. Prostacyclin had been discovered by John Vane, then a prominent pharmacologist at Welcome laboratories in the United Kingdom who was interested in supporting studies of prostacyclin as a therapeutic agent for a host of diseases. While most of those studies failed to provide evidence of a therapeutic potential for prostacyclin, we were able to demonstrate that acute and short‐term administration of prostacyclin produced beneficial effects in a series of PPH patients [4]. Tim Higenbottam at Cambridge took the next step by showing that ambulatory continuous intravenous prostacyclin was feasible and improved hemodynamics in several PPH patients [5]; this was followed by Phase 2 and 3 studies [6, 7] that led to the approval of prostacyclin (Epoprostenol) for the treatment of PAH‐ the first drug approved for this condition and still the go‐to therapy for the most seriously ill patients.

Several months after Mr. C's death, I was visited by investigators from the North Carolina Bureau of Investigation who were looking into his death, which they called “suspicious.” They told me that Mr. C had owned a small parcel of farmland that was in the middle of a federally planned interstate highway and he had been offered a substantial sum to sell the plot to the government. Mr. C had stubbornly refused, saying that the land had been in his family since the end of the Civil War and he was unwilling to break the inheritance chain. Mrs. C, however, had other plans; given Mr. C's health, she wanted the money now to provide for her life once her husband was no longer around. But time was of the essence. So, she had put arsenic in her husband's food for several months until he finally died. The body was exhumed, and an autopsy confirmed his cause of death as severe arsenic poisoning! In retrospect, his symptoms over the few months before his death were typical for arsenic poisoning, including the unusual anemia he had developed. Mrs. C, the caring, loving, doting spouse whom I consoled so empathetically after Mr. C's death, was convicted of his murder and sent to prison for life.

Since then, I've cared for many more patients with pulmonary hypertension. The path has been challenging yet gratifying, even though the goal of a cure has remained elusive. I learned a lot from my first PPH patient, and I continue to learn to this day from both him and other patients whom I've cared for over the past 50 years.

## Author Contributions

The author is solely responsible for the contents of this article.

## Ethics Statement

Patient confidentiality protected.

## Conflicts of Interest

The author declares no conflicts of interest.
